# Transcutaneous auricular vagus nerve stimulation in disorders of consciousness: A mini-narrative review

**DOI:** 10.1097/MD.0000000000031808

**Published:** 2022-12-16

**Authors:** Sung Ho Jang, Min Jye Cho

**Affiliations:** a Department of Physical Medicine and Rehabilitation, College of Medicine, Yeungnam University, Namku, Taegu, Republic of Korea.

**Keywords:** brain injury, consciousness, disorders of consciousness, recovery, transcutaneous auricular vagus nerve stimulation

## Abstract

In this mini review, 6 studies that investigated the effects of transcutaneous auricular vagus nerve stimulation (taVNS) in patients with disorders of consciousness (DOC) were reviewed. Generally, the application of taVNS in patients with DOC appears to be effective (positive results in 5 of 6 studies) and safe. Furthermore, 4 studies that evaluated changes in the brain following taVNS reported positive results (2 studies, functional magnetic resonance imaging and 2 studies, electroencephalography). Based on our review of the 6 studies, we believe that research and clinical application of taVNS in DOC are in the initial stages and have the following limitations. First, there is a shortage of studies on this topic, with only 6 studies, 2 of which were case reports. Second, 5 studies were performed without control or sham groups. Third, there was no standardization of treatment schedules and electrical stimulation parameters. Therefore, further studies to overcome the above limitations should be encouraged; further original studies involving a larger number of patients in the control or sham groups are needed. However, studies on the optimal conditions (treatment schedule and electrical stimulation parameters) for taVNS in patients with DOC are necessary. Furthermore, neuroimaging studies should be undertaken to elucidate the neurological mechanisms for the recovery of impaired consciousness in DOC and the lasting effects of taVNS on the brain.

## 1. Introduction

Disorders of consciousness (DOC) frequently follow severe brain injuries, including traumatic brain injury (TBI), stroke, and anoxic brain injury. In recent years, neuromodulatory therapies for DOC have been widely used and are classified as invasive or noninvasive based on the need for surgical assistance.^[1–3]^ noninvasive neuromodulatory therapies are more commonly used than invasive therapies because they offer the advantages of convenience, safety, and cost-effectiveness.^[2,3]^ These therapies comprise transcranial direct current stimulation, repetitive transcranial magnetic stimulation, transcranial focused ultrasound pulsation, and transcutaneous vagus nerve stimulation (VNS).^[1–3]^ VNS is a nerve stimulation technology that modulates functional brain activity through electrical stimulation of the vagus nerve.^[4–7]^ Compared to transcranial direct current stimulation and repetitive transcranial magnetic stimulation, transcutaneous VNS is still in the nascent stages of research and clinical application.^[1–7]^ Transcutaneous VNS is classified based on the choice of afferent branches of the vagal nerve used for stimulation. These include transcutaneous auricular VNS (taVNS), involving the auricular branch of the vagus nerve, and transcutaneous cervical VNS, involving the cervical branch of the vagus nerve in the neck.^[1,2,4,5,7]^ taVNS is more commonly used than transcutaneous cervical VNS owing to its ease of application.^[1,2,4,5,7]^

The vagus nerve (the 10th and longest cranial nerves) is the strongest parasympathetic nerve in the autonomic nervous system.^[8,9]^ It is a mixed nerve composed of 20% efferent fibers and 80% afferent fibers and serves as a bidirectional communicator between the brain and body.^[8,9]^ The auricular branch of the vagus nerve provides somatosensory innervation to the skin of the ear canal, tragus, and auricle. The cymba conchae in the auricle are considered the best locations for taVNS because they are completely innervated by the auricular branch of the vagus nerve.^[5,8,10]^ The afferent fibers of the auricular branch are connected to the nucleus of the solitary tract through the spinal trigeminal nucleus.^[5,8,11–13]^ The solitary tract nucleus projects to the dorsal motor nucleus and nucleus ambiguus, and regulates the central autonomic activity of the body.^[5,8,14,15]^ This provides the neurological basis for the use of taVNS in the treatment of diseases or disorders of the body.^[5,8,14,15]^

Many studies have also reported that taVNS modulates or activates the cerebral cortices and subcortical areas that are related to the control of consciousness, including the locus coeruleus, raphe nuclei, thalamus, striatum, hippocampus, parahippocampus, hypothalamus, medial prefrontal cortex, orbitofrontal cortex, dorsolateral prefrontal cortex, anterior cingulate cortex, posterior cingulate cortex, precuneus, parietal cortex, and temporal cortex.^[8,9,15–35]^ This is the neurological basis for exploring the therapeutic potential of taVNS to modulate levels of consciousness.^[20,23–25,28,33,35]^ Since the first study by Yu et al in 2017, 6 studies on the application of taVNS in patients with DOC have been reported.^[23,28,33,35–37]^

In this mini-review article, we reviewed 6 studies that investigated the effects of taVNS in patients with DOC.^[23,28,33,35–37]^

### 1.1. Brain networks involved in the control of consciousness

**Figure 1.**
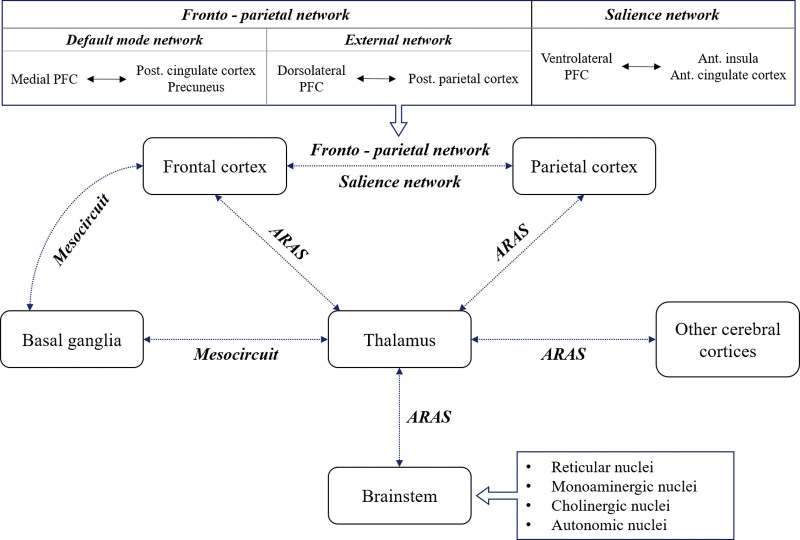
Diagram of neural networks for the control of consciousness. ARAS: ascending reticular activating system, PFC: prefrontal cortex.

The neural mechanisms involved in the control of consciousness in the human brain have not yet been clearly elucidated. However, complicated and interactive neural networks through which various regions of the brain interact have been suggested to be responsible for the control of consciousness (Fig. 1).^[24,38–61]^ The ascending reticular activating system (ARAS), which originates from the reticular nuclei in the upper brainstem and connects with the central thalamus (the intralaminar nuclei and related paralaminar nuclei) and cerebral cortex, is involved in wakefulness and vigilance.^[24,45,50,53,55,60]^ The ARAS provides neuronal inputs to the anterior forebrain mesocircuit (cortico-striatal-thalamic-cortical loop) and the frontoparietal network to activate cortical neurons.^[24,45,47,60]^ The frontal lobe and central thalamus are closely linked through the thalamocortical tract, directly and indirectly through the mesocircuit model.^[24,38,39,45,48,60]^ The mesocircuit model provides a concept for the role of the central thalamus in neuromodulation to support forebrain arousal regulation.^[24,58,60]^ The anterior forebrain mesocircuit and frontoparietal network are strongly interconnected in the mesocircuit–frontoparietal model.^[24,57,60]^ The frontoparietal network comprises 2 subnetworks: the internal default mode network (DMN) and the external network (EXN, external frontoparietal network, executive control network, and task-positive network).^[24,52,56,60]^ The DMN connects the frontal (medial prefrontal cortex and anterior cingulate cortex) and posterior parts (posterior cingulate cortex, precuneus, retrosplenial cortex, inferior parietal lobule, temporal lobe, and part of the hippocampal formation) of the brain and mediates internal awareness or self-related processes.^[24,40,44,49,51,56,59]^ In contrast, the EXN, which connects the dorsolateral prefrontal and posterior parietal cortices, mediates attention, action selection, and the selection of relevant environmental information.^[24,41–43,56,60]^ Fox et al suggested a negative connectivity between the DMN and EXN, which means a negative correlation between the 2 networks, and that activation of the EXN is associated with a synchronized decrease in activation of the DMN.^[24,40,43]^ The salience network (SN), which connects the ventrolateral prefrontal cortex, anterior insula, and dorsal anterior cingulate cortex, is known to be involved in switching between the EXN and DMN.^[24,42,43,46,54]^

In their review, Briand et al^[24]^ have elaborated on the key brain areas and networks of consciousness. These include:

(1) Active key brain areas: 4 brainstem nuclei

i. The reticular nucleus: cuneiform, deep mesencephalic nucleus, part of the pedunculopontine tegmental nucleus, and pontis oralis nucleus.
ii. monoaminergic nuclei: locus coeruleus [norepinephrine], raphe nuclei [serotonin], substantia nigra, and ventral tegmental area [dopamine].
iii. cholinergic nuclei: pedunculopontine and laterodorsal tegmental nuclei.
iv. Autonomic nuclei: parabrachial nucleus and periaqueductal gray matter, thalamus, and posterior cingulate cortex.

(2) within the DMN and EXN connectivity and negative connectivity between the DMN and EXN, which appear to be controlled by the SN.

(3) an intact mesocircuit model.^[24]^

The above key brain areas and neural networks can thus be targets for inducing the recovery of impaired consciousness in patients with DOC.^[24,60]^

### 1.2. Vagal cortical pathways model for DOC

**Figure 2.**
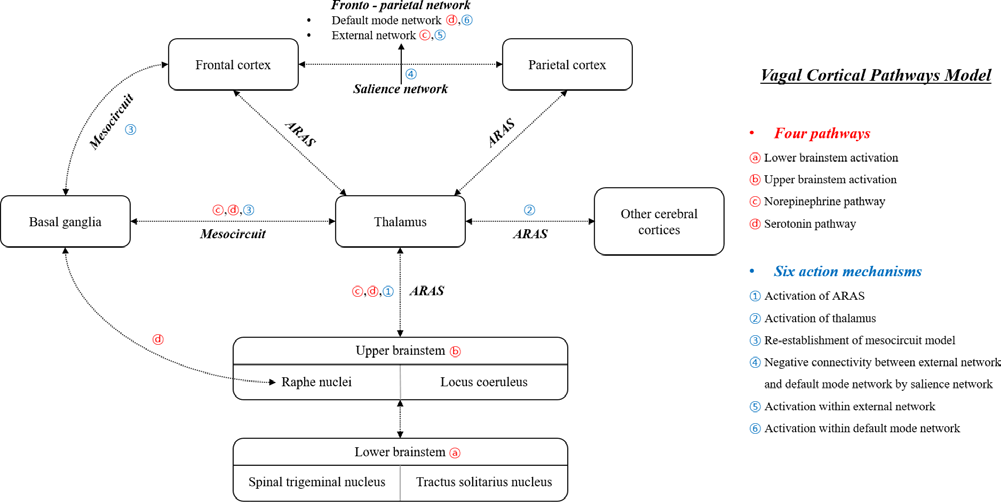
Diagram of the vagal cortical pathways model proposed by Briand et al^[24]^. ARAS = ascending reticular activating system.

In recent years, several studies have reported the therapeutic potential of taVNS in the recovery of impaired consciousness in patients with DOC.^[23,28,33,35–37]^ However, the therapeutic mechanisms of this recovery have not been clearly elucidated to date.^[24]^ To explain the potential therapeutic mechanisms, Briand et al^[24]^ proposed the vagal cortical pathways model comprising 4 pathways: lower brainstem activation, upper brainstem activation, norepinephrine pathway, and serotonin pathway, and 6 action mechanisms: activation of the ARAS, activation of the thalamus, reestablishment of the mesocircuit model (cortico-striatal-thalamic-cortical loop), promotion of negative connectivity between the EXN and DMN by activation of the salience network, activation within the EXN through the norepinephrine pathway, and activation within the DMN through the serotonin pathway (Fig. 2).

TaVNS induces the activation of the spinal trigeminal nucleus through the auricular branch of the vagus nerve and subsequently the tractus of the solitarius nucleus located in the lower brainstem (pathway A).^[5,8,11–13]^ These nuclei activate the locus coeruleus and the raphe nuclei located in the ARAS of the upper brainstem (pathway B) and participate in the arousal (mechanism 1).^[13,15,62,63]^ The 2 activated nuclei further lead to the activation of the subcortical areas and neurotransmitter pathways; the locus coeruleus (norepinephrine pathway) and the raphe nuclei (serotonin pathway) activate the thalamus directly (mechanism 2, and pathways C and D).^[5,13,15,20]^ The thalamic activation induces the activation of the striatum, which is also directly connected to the raphe nuclei, and consequently, facilitates the reestablishing of the mesocircuit model (mechanism 3).^[45,47,48]^ The activation of the locus coeruleus which is the norepinephrine pathway may influence consciousness by promoting the activation of the SN and EXN (mechanisms 4 and 5).^[64–66]^ The activation of the SN facilitates switching from the DMN to the EXN which could improve the negative connectivity between the DMN and EXN (mechanism 4).^[24,42,43,46,54]^ The activation of the raphe nuclei from which the serotonin pathway originates can increase the activity and connectivity of the DMN (pathway C and mechanism 6).^[15,24,67]^

### 1.3. Clinical data on taVNS in DOC

In 2017, Yu et al reported the case of a patient who showed partial recovery of impaired consciousness from a vegetative state (VS) to a minimally conscious state (MCS) after 4 weeks of taVNS (Table 1).^[23]^ A 73-year-old female patient presented with respiratory and cardiac arrests. Although immediate cardiopulmonary resuscitation was performed, her impaired consciousness did not improve until 50 days after onset and was diagnosed with VS.^[23]^ TaVNS was applied to the patient’s bilateral ear concha for 4 weeks commencing 50 days after onset (30 min/session, twice/d, frequency: 20 Hz, wave width; <1 ms, and intensity: 4–6 mA). Her impaired consciousness recovered from 6 points (VS) to 13 points (MCS, new behaviors in both motor and oromotor functions) on the JFK Coma Recovery Scale-Revised (CRS-R) after 4 weeks of TaVNS.^[68–70]^ Functional magnetic resonance imaging was performed twice before and after 4 weeks of taVNS treatment to determine the changes in functional connectivity in the DMN following taVNS treatment. With the seed region in the posterior cingulate cortex, the functional connectivity between the posterior cingulate/precuneus, hypothalamus, thalamus, ventral medial prefrontal cortex, and superior temporal gyrus increased, whereas the functional connectivity between the posterior cingulate/precuneus and cerebellum decreased. The authors concluded that taVNS treatment induced increased functional connectivity of the DMN in this patient, which could be the primary reason for the recovery of impaired consciousness.^[24,40,44,49,51,56,59]^ This is the first study to report recovery of impaired consciousness after taVNS treatment in patients with DOC. However, the possibility of coincidental spontaneous recovery of impaired consciousness could not be excluded because there were no control subjects, a fact that was also mentioned by the authors.^[23]^

**Table 1:** Summary of studies on transcutaneous auricular vagus nerve stimulation in disorders of consciousness.

Publication	Patients no.	Brain pathology	taVNS device	Stimulation side and site	Stimulation parameter			Data cycle			Clinical results	Side effect	Brain evaluation
					Hz	Pulse width (us)	Intensity (mA)	Min/session	Time/d	Period			
Yu et al^[23]^ (2017)	1	Anoxia	NC	Bilateral cymba conchae	20	1000	4~6	30	2	50 days	CRS-R: 6→13	NC	fMRI: DMN connectivity↑
Noé et al^[36]^ (2020)	14	TBI: 7 Anoxia: 4 Hemorrhage: 3	Parasym®	Left tragus	20	250	1.5	30	2	4 weeks	5 of 8 MCS patients: CRS-R↑	None	-
Hakon et al^[37]^ (2020)	5	TBI	Nemos®	Left cymba conchae	25	250	0.5~1	240	1	8 weeks	All MCS (2)→emerged from MCS 1 of 3 VS→MCS	Intermittent itching of the ear (1 patient)	-
Yu et al^[28]^ (2021)	10	Anoxia: 5 Hemorrhage: 3 TBI: 2	NC	Cymba conchae	20	500	4~6	30	2	4 weeks	Responded to auditory stimuli (5): CRS-R↑& favorable outcome	NC	fMRI: Auditory responded group: CBF↑of multiple brain regions
Osińska et al^[33]^ (2022)	1	TBI	Nemos®	Cymba conchae	25	250	0.2→1.5	30	2	6 months	CRS-R: 4~6→8~13	NC	EEG: alpha range↑
Yifei et al^[35]^ (2022)	12	Stroke: 8 Anoxia: 2 TBI: 2	Huatuo electronic acupuncture	Bilateral cymba conchae	20	1000>	4~6	30	2	14 days	CRS-R: no change	NC	EEG: MCS patients in taVNS group: Delta band↑ and Beta band↓

CBF: cerebral blood flow, CRS-R: Coma Recovery Scale-Revised, EEG: electroencephalography, fMRI: functional magnetic resonance imaging, DMN: default mode network, MCS: minimally consciousness state, NC: not commented, TBI: traumatic brain injury, taVNS: transcutaneous auricular vagus nerve stimulation, VS: vegetative state.

Noe et al^[36]^ investigated the feasibility, safety, and therapeutic effects of the taVNS treatment in 14 patients with DOC following brain injury (TBI, 7 patients; anoxia, 4 patients and hemorrhage, 3 patients). Of the 14 patients (40.2 ± 16.1 years) with DOC, 6 patients were in a VS and 8 were in an MCS more than 6 months after the brain injury (12.1 ± 6.4 [6–27] months after onset). These 14 patients showed no changes in their CRS-R scores, checked weekly in the 4 weeks preceding taVNS treatment.^[36]^ TaVNS (Parasym® CE) was applied to the left tragus for 4 weeks (30 min/session, twice/day, 5 d/wk, sinusoidal waveform, frequency; 20 Hz, pulse width; 250 us, amplitude; 1.5 mA).^[23]^ The CRS-R was evaluated at baseline (T0), week 1 (T1), week 2 (T2), week 3 (T3), and week 4 (T4: end of treatment) with a further follow-up 4 weeks after the termination of taVNS. The CRS-R scores significantly increased at the end of the one-month follow-up. However, none of the VS patients presented any change in the CRS-R scores, while 5 of the 8 MCS patients revealed an incremental increase of the CRS-R during this study; the CRS-R increased in only one MCS patient at the end of the treatment (T4) and this patient and 4 more MCS patients showed an increase in the CRS-R at the 1-month follow-up (T4 + 4). Four patients had an increase in only one CRS-R subscale (motor subscale: 3 patients and visual subscale: 1 patient), while one patient showed an increase in more than one CRS-R subscale (including the motor subscale).^[68–70]^ Although 8 mild adverse effects were reported from a total of 560 sessions performed, all these were considered common medical conditions unrelated to the taVNS. No relevant changes were observed in the echocardiogram, heart rate, and blood pressure. The authors concluded that taVNS is a feasible and safe option for patients with DOC and it may improve behavioral responses in patients with MCS. This was the first study to demonstrate feasibility, safety, and efficacy in patients with DOC. However, the weakness of this study was the lack of a control group and the heterogenous brain pathologies of the patients included in the study.

Hakon et al^[37]^ investigated the feasibility and safety of taVNS in patients with persistent impairment of consciousness following severe TBI. Five adult patients (mean age 67 years [range, 21–80 years]) were diagnosed with persistent VS or MCS for more than 28 days after diffuse axonal injury (VS, 3 patients and MCS, 2 patients) and reduced dominant electroencephalography (EEG) activity at 1 month after onset. TaVNS (Nemos®, Cerbomed, Germany; CE-marked 2011) was applied to the left cymba conchae for 8 weeks (once/day, 4 h/session, pulse width: 250 µs, frequency: 25 Hz, 30 s on/ 30 s off, amplitude; up to 0.5 mA for the first 3 days and subsequently 1 mA for the remaining period). No patient presented with any signs of discomfort, including signs of pain/nociception, grimacing, diaphoresis, or any other symptoms of sympathetic or parasympathetic overdrive. Furthermore, taVNS had no effect on blood pressure, pulse rate, and mean arterial pressure. Although 1 patient showed intermittent itching of the ear during stimulation, this was not to a degree that required a decrease in the quantum of stimulation. Three patients showed improvement (>3 points) in the CRS-R after 8 weeks of taVNS. Furthermore, 2 MCS patients emerged from it, and one VS patient progressed to MCS during the 8 weeks of taVNS.^[68–70]^ Consequently, the authors concluded that taVNS is a feasible and safe strategy for patients with DOC after severe TBI. However, the absence of control or sham groups is an important limitation of this study. Furthermore, the recruited patients seemed to be in the recovery phase of impaired consciousness, although the authors insisted that they included patients with persistent VS or MCS because the recovery phase of diffuse axonal injury is 1 year after onset.^[71]^

Yu et al^[28]^ investigated the treatment efficacy and cerebral hemodynamic changes of taVNS in patients with DOC. Ten patients (19–73 years old) with DOC following severe brain damage after acute brain injury (anoxia, 5 patients; hemorrhage, 3 patients; and TBI, 2 patients) for at least 2 days (10–300 d) after onset were included in the study. TaVNS was applied to the concha area (the cymba concha and cavity of the concha) for 4 weeks (30 min/session, twice/d, frequency: 20 Hz, pulse width: 500 µs, amplitude: 4–6 mA). CRS-R and functional magnetic resonance imaging were performed twice: before and after taVNS treatment. Before taVNS, 5 patients responded to auditory stimuli (RtAS group, auditory subscale of the CRS-*R* ≥ 1: auditory startle) and 5 did not respond to auditory stimuli (nRtAS group, auditory subscale; 0). CRS-R significantly increased in the RtAS group, while no significant change was observed in the nRtAS group.^[69]^ Furthermore, the patients in the RtAS group showed a favorable outcome on the Glasgow Outcome Scale after the 4-week taVNS treatment, whereas the nRtAS patients showed unfavorable outcomes.^[72]^ Simultaneously, in the RtAS group, taVNS increased cerebral blood flow in multiple brain regions (the superior temporal gyrus, left prefrontal cortex, medulla, cerebellum, precentral gyrus, right caudate, right hippocampus, left insula, left occipital cortex, and right thalamus), whereas the increase in cerebral blood flow in the nRtAS group was relatively weak with taVNS treatment and was prominent only in the left cerebellum. The authors concluded that preserved auditory function may be an important factor in achieving the clinical benefit of taVNS treatment in patients with DOC. A limitation of this study was the absence of a control group. In addition, before taVNS, the patients in the RtAS group were clinically better than those in the nRtAS group. Three patients in the RtAS group were in an MCS and 2 were in a VS, whereas all 5 of the nRtAS subjects were in a VS.

In 2022, Osińska et al^[33]^ reported the case of a patient who showed recovery of impaired consciousness after 6 months of taVNS treatment. A 28-year-old woman was diagnosed with persistent VS at 4 points on the CRS-R following TBI which occurred 6 years previously.^[33]^ TaVNS (NEMOS^R^ stimulator, tVNS Technologies, Erlangen, Germany) was applied to the cymba concha for 6 months (30 min/session, twice/day, monophasic square wave, pulse width; 250 us, frequency; 25 Hz, amplitude; 0.2 mA →1.5 mA [increasing the intensity by 0.1 mA every week up to 1.5 mA]). The consciousness state was assessed using the CRS-R scale, just prior to the taVNS treatment, weekly during the 6-month taVNS treatment, and 9 weeks after the end of the taVNS treatment. From the pre-taVNS treatment score of 4–6 points, the patient’s CRS-R improved to 8–10 points after approximately 100 days of taVNS treatment, and occasionally the CRS-R score increased to 13 points. These results indicated that her status changed from VS to MCS or even MCS+.^[68,69]^ However, the fact that the CRS-R scores after taVNS treatment were lower than the maximum CRS-R score during taVNS treatment suggested that the treatment might not have a lasting effect. Among the CRS-R subscales, the auditory and visual subscales had the greatest impact on CRS-R improvement. These results indicated that the behavioral changes caused by taVNS treatment in this patient were closely related to visual and auditory functions. The EEG frequency spectrum profiles showed the reemergence of a second oscillatory peak in the alpha range, which has been shown to characterize “aware” people, and sustained spontaneous theta oscillations did not predictably diminish, most likely reflecting structural brain damage. Second, over the course of several months of taVNS treatment, the EEG power in the alpha wave range gradually increased, which may be an indicator of marginal neural network reintegration and strengthening of cortical activity.^[25,73]^ Electrocardiography revealed a steady decrease in the pre-stimulation heart rate combined with an increase in heart rate variability (HRV). This suggests a gradual withdrawal of the sympathetic nervous system and an increase in the parasympathetic control of the heart, which prior literature has also linked with improvements in DOC. The resting HR gradually decreased, and the high-frequency rate variability (HRV-HF) increased. This may be a sign of the increasing influence of the parasympathetic system in the autonomic space, which usually helps to improve environmental consciousness. The advantage of this study was that the authors demonstrated the changes due to taVNS using 3 measurements: behavioral changes seen through changes in CRS-R, changes in cortical activity observed on EEG, and changes in the autonomic nervous system confirmed through changes in HR and HRV-HF. A limitation of this study was that it was a case study without control or sham subjects.

Yifei et al^[35]^ investigated the effect of taVNS in 12 patients (mean age; 36.5 ± 10.8; range, 18–53 yr and 3–13 months after onset) with DOC (VS,7 patients and MCS, 5 patients) due to acquired brain injury (stroke, 8 patients; anoxia, 2 patients and TBI, 2 patients). These patients were assigned to 2 groups: taVNS or transcutaneous nonauricular VNS (tnVNS). VNS (Huatuo; electronic acupuncture instrument, SDZ-II B type, Suzhou Medical Products Factory Co., Ltd.) was applied for 14 days (30 min/session, twice/day, pulse width; <1ms, frequency: 20 Hz, amplitude: 4–6 mA). VNS was applied to the bilateral auricular concha in the taVNS group and to the bilateral auricular nonconcha region (the tail of the helix, which is thought to be free of vagal innervation) in the tnVNS group. Furthermore, the patients were divided into the VS and MCS groups. All patients in the taVNS and tnVNS groups failed to show any significant improvement on the CRS-R scale. The resting state EEG power spectrum revealed a decrease in the energy of the delta band and an increase in the energy of the beta band in MCS patients in the taVNS group, which coincided with the results of previous studies and indicated better consciousness, while contrasting results were seen in the VS patients in the taVNS group.^[74–76]^ No significant changes were observed in the tnVNS group. The authors concluded that taVNS could be a possible treatment for patients with DOC, and that the effects might be more remarkable in MCS patients than in VS patients. The major limitation of this study was that there was no clinical improvement in any patient despite the EEG changes. The authors stated that the short treatment period of 14 days and lack of follow-up data were the limitations of this study.

## 2. Conclusions

In this mini review, 6 previous studies that investigated the effects of taVNS in patients with DOC were reviewed.^[23,28,33,35–37]^ Overall, the application of taVNS in patients with DOC appeared to be effective (positive results seen in 5 out of 6 studies)^[23,28,33,36,37]^) and safe (only one of 43 patients presented with an itching sensation in the ear^[37]^). Furthermore, 4 studies that estimated changes in the brain following taVNS reported positive results (2 studies functional magnetic resonance imaging^[23,28]^ and 2 studies EEG^[33,35]^). Regarding the application methods, the application site and time schedules were similar (5 studies: cymba conchae, 30 min/session and 2 times/d), whereas the treatment period was quite variable (14 d to 6 mo). With regard to the electrical stimulation parameters, the frequency was similar in all studies at 20–25 Hz; however, the pulse width (200–1000 µs) and intensity (0.2–6 mA) were variable. Based on our review of the 6 studies, we believe that research on taVNS in DOC is still in the nascent stages and has the following limitations. First, there is a paucity of clinical data on this topic; only 6 studies, and 2 of the 6 studies were case reports.^[23,28,33,35–37]^ Second, 5 studies were performed without control or sham groups.^[23,28,33,36,37]^ Third, there was no standardization of treatment schedules and electrical stimulation parameters. Therefore, further studies to overcome the above limitations should be encouraged, and original research studies involving a larger number of patients with control or sham groups are needed. However, studies to optimize the treatment parameters (time schedule, treatment period, and electrical stimulation parameters) of taVNS for patients with DOC are necessary.^[16–18]^ Furthermore, relevant neuroimaging studies should be encouraged to elucidate the neurological mechanisms involved in the recovery of impaired consciousness in DOC and the lasting effects of taVNS on the brain.

## Author contributions

**Conceptualization:** Sung Ho Jang, Min Jye Cho.

**Data curation:** Sung Ho Jang, Min Jye Cho.

**Investigation:** Sung Ho Jang, Min Jye Cho.

**Supervision:** Sung Ho Jang.

**Visualization:** Min Jye Cho.

**Writing**—**original draft:** Min Jye Cho.

**Writing—review and editing:** Sung Ho Jang.
